# Behavior Change and Compliance Outcomes of a Novel Web-Based Health Behavior Tool Integrated Into Certified Exercise Practitioner Services: Twelve-Week, Two-Arm, Randomized Pre-Post Trial

**DOI:** 10.2196/90115

**Published:** 2026-09-30

**Authors:** Kade Davison, Anthony Mezzini, Nathan Harten, Max Martin, David Lowrey, Holly Evans, Janis Onuzans, William Bastian, Rohan Singh Howard, Bethany Gower

**Affiliations:** 1 Alliance for Research in Exercise, Nutrition and Activity, School of Allied Health and Human Performance, College of Health, Adelaide University, Adelaide, South Australia, Australia; 2 School of Allied Health and Human Performance, College of Health, Adelaide University, City East Campus, Playford Building P4-27, North TerraceAdelaide, South Australia, 5000, Australia, 61 0427820256; 3 iNform Health and Fitness Solutions, Adelaide, South Australia, Australia; 4 College of Nursing and Health Sciences, Flinders University, Adelaide, South Australia, Australia; 5 Allied Health and Human Performance, University of South Australia, Adelaide, South Australia, Australia

**Keywords:** digital health, lifestyle behaviors, clinical exercise practitioners, behavior change, web-based intervention

## Abstract

**Background:**

Healthy lifestyle behaviors, including a balanced diet, regular physical activity, adequate sleep, avoiding tobacco, moderating alcohol consumption, and stress management, are associated with reduced risk of chronic disease and improved well-being. In recent years, digital interventions have emerged as cost-effective platforms for promoting these behaviors, yet few have been integrated into services delivered by certified exercise practitioners (CEPs) or focused on multiple domains of health.

**Objective:**

This study aimed to examine changes in health behaviors during implementation of the Food, Exercise, Sleep, and Stress (FESS) web-based questionnaire as an adjunct to traditional face-to-face services provided by CEPs and compare these changes across 2 prompting frequencies.

**Methods:**

A 2-arm, randomized pre-post intervention design was conducted with 41 participants (23 women), with a median age of 65 (IQR 58‐70) years, who were recruited from CEP clinics. Participants were randomized to complete the FESS questionnaire either 3 times per week (3-day group, n=21) or daily (everyday group, n=20) for 12 weeks. The questionnaire tracked health behaviors across 4 domains and provided health tips. Outcomes were assessed using the assessment version of the FESS questionnaire at baseline and at 6 and 12 weeks. Changes in FESS questionnaire responses were examined over time using Friedman ANOVA with Wilcoxon signed-rank post hoc tests. Group differences between prompting frequencies were assessed by calculating changes in questionnaire responses from baseline to 6 and 12 weeks and analyzing them using the Mann-Whitney *U* test. Compliance was assessed by calculating the proportion of completed FESS questionnaires relative to the total number of questionnaires prescribed for each participant within each intervention arm. Validity of the exercise domain was evaluated by comparing self-reported physical activity and sedentary time from daily FESS responses with accelerometer-derived measures in a subsample of participants.

**Results:**

Positive or no changes were observed across most behaviors, with significant improvements in vegetable consumption, water intake, stress, and sleep. Group comparisons indicated larger changes in stress and sleep behaviors in the everyday group; however, compliance was higher in the 3-day group. Compliance declined over time in both groups. Accelerometer validation of the exercise domain showed low rates of overreporting exercise (3/59, 5.1%) and underreporting sedentary behavior (11/59, 18.6%).

**Conclusions:**

The FESS questionnaire demonstrated feasibility and was associated with observed changes in health behaviors, particularly in diet, stress, and sleep, when integrated into CEP services. While daily use was associated with larger observed changes, reduced compliance highlights the importance of flexible engagement strategies. These preliminary findings suggest that CEP services may benefit from embedding structured digital tools into traditional face-to-face interactions, as such tools may support education, promote reflection and accountability between sessions, and facilitate attention to a broader range of lifestyle factors within routine CEP care. Further controlled studies are required to confirm these findings.

## Introduction

Healthy behaviors refer to actions and lifestyle choices that promote physical and mental well-being. They include regular physical activity, a balanced diet, adequate hydration, avoiding tobacco use, maintaining a healthy body weight, moderating alcohol and caffeine consumption, adequate sleep, reduced sitting and screen time, and managing stress [1-4]. The impact of healthy behaviors on morbidity and mortality is well documented. Research evidence has linked positive health behaviors to reduced rates of chronic diseases such as cancer, heart disease, stroke, diabetes, and dementia [5-10], while also contributing to improved mental health, well-being, and cognitive function [11,12]. Furthermore, a healthy lifestyle has been associated with an estimated increase of more than 10 years in life expectancy in several developed countries, such as Japan [13], the United States [14], and the United Kingdom [15]. As a result, promoting healthy behaviors has become a cornerstone of public health strategies aimed at reducing the global burden of disease and enhancing quality of life [16].

Health behavior promotion interventions commonly incorporate one or more of the following components: educational initiatives to increase awareness of health concerns, motivational strategies and goal setting, and community-based techniques that encourage behavior change or address structural or cultural barriers [3]. These interventions can be implemented at the individual, community, or population levels and can be delivered through a variety of channels, including traditional face-to-face, print, mass media, and digital platforms [17]. In recent years, digital interventions have emerged as a low-cost and convenient platform for delivering personalized educational, motivational, and goal-setting strategies directly to individuals [18-20]. Importantly, research has demonstrated that digital health promotion interventions have been effective in improving several healthy behaviors and lifestyle factors, including weight management, smoking cessation, physical activity levels, alcohol consumption, and self-management of chronic diseases [21-24].

In parallel, certified exercise practitioners (CEPs) also play a role in delivering behavior change interventions, typically through traditional face-to-face platforms at the individual or group level [25,26]. While the benefits of these programs have been documented in the literature [27], several barriers have also been identified, including inconsistencies in practitioner knowledge and skills [25,28,29] and out-of-pocket costs for accessing CEP-delivered services, particularly among individuals from lower socioeconomic groups who are at greater risk of poor health [30]. Combining digital health promotion interventions with traditional face-to-face programs delivered by CEPs may be a convenient and cost-effective way to leverage the benefits of both approaches while addressing their respective limitations [31].

The Food, Exercise, Sleep, and Stress (FESS) questionnaire is a web-based behavior change tool developed by an Australian CEP clinic that has been available to its clientele for the past 6 years. It is designed to be accessed regularly (3-7 days a week) and promotes short- and long-term accountability and encouragement by tracking healthy behaviors while also providing practical health advice and tips across 4 domains: food, exercise, stress, and sleep. Clients who complete the FESS questionnaire are encouraged to discuss the results with their CEP to identify strengths and areas for improvement. The questionnaire’s features align with established behavior change techniques, such as self-monitoring, feedback on behaviors, instruction, knowledge, and repetition [32]. The extent to which the FESS questionnaire is associated with subsequent changes in health behaviors has not yet been established. The primary aims of this study, therefore, were to evaluate changes in health behaviors following implementation of the FESS tool within existing CEP service and to compare these changes across 2 prompting frequencies. The secondary aims were to compare engagement (adherence or compliance) across prompting frequencies and to assess the reporting accuracy of the exercise domain of the FESS questionnaire.

## Methods

### Methodology

This study used a 2-arm, randomized pre-post intervention design. The study protocol was reported in accordance with the CONSORT (Consolidated Standards of Reporting Trials) 2010 statement: extension to randomized pilot and feasibility trials, which provides a framework for transparent reporting of small-scale randomized studies with exploratory outcomes [33] (Checklist 1).

### Sample and Recruitment

People accessing CEP services across various sites in South Australia were recruited for this study. Recruitment sites included the Queen Elizabeth Hospital, Hampstead Rehabilitation Centre, GP Plus Healthcare Centre Elizabeth, the University of South Australia Exercise Physiology Clinic, iNform Health and Fitness Solutions, and Fleurieu Exercise Physiology. A target sample size of 40 (20 per arm) was deemed sufficient based on results from previous studies using similar interventions [21,34]. Eligibility criteria included being a newly referred or current CEP service client and proficiency in reading and writing English. People without access to an internet-enabled device, or with insufficient IT skills or cognitive function to operate a web-based questionnaire, were not eligible to participate.

Convenience sampling with active recruitment was used to recruit participants [35]. The study was promoted to potential participants through face-to-face interaction with researchers and CEP staff at participating sites, as well as via email. Individuals who expressed interest were contacted by the research team and screened for eligibility. Those who met the eligibility criteria were provided with a Participant Information Sheet and asked to provide informed written consent. They were then asked to complete a brief questionnaire, either in hard copy or online via REDCap (Vanderbilt University), capturing demographic (age and sex) information and CEP service client status (current or newly referred).

### Instrument

The FESS questionnaire was developed by members of the research team (MM and NH), both CEPs, and was informed by over 30 years of combined clinical experience as well as existing literature on lifestyle-related health behaviors [1-4] and behavior change principles [3,17-24]. Items were generated to reflect key modifiable lifestyle domains addressed in CEP practice, including exercise as a key domain, alongside related areas, such as diet, stress management, and sleep. These additional domains, while not a key focus, are often considered in practice because they influence clients’ ability to engage in physical activity and achieve overall health outcomes.

The questionnaire was designed as a pragmatic clinical tool to support behavioral reflection and tailored feedback within the intervention, rather than as a psychometrically validated, domain-specific instrument. The questionnaire comprises 23 items. Items 1 to 8 of the questionnaire address food-related behaviors, items 9 to 12 focus on exercise, items 13 to 18 focus on stress, and items 19 to 23 focus on sleep. The response options are predominantly categorical (22 items), with only 1 item using a numerical response format. Each response option is assigned a numerical score; these are then summed to produce a total score out of 100, with higher scores reflecting healthier behaviors. As respondents progress through the questionnaire, they are presented with health advice and tips specific to each item and its associated health domain. For example, within the exercise domain, respondents are provided with guidance such as: “The amount, type, and intensity of your exercise, along with your sedentary behaviors, are strong predictors of health. A combination of strength and aerobic exercise, together with breaking up prolonged sitting, are key behaviors that support health.” Within the stress domain, advice includes messages such as: “It’s not only the amount of stress you are under but how you react to it which influences your health. Developing behaviors to improve resilience have been shown to influence longevity. They can also impact our motivation to exercise as well as influence our food choices.”

### Intervention

The intervention was conducted between August and December 2022. Participants were randomly allocated to 2 groups using block randomization to ensure balanced distribution by age, sex, and whether they were a newly referred or current CEP service client. One group was required to complete the FESS questionnaire 3 times per week (3-day group), while the other group completed the FESS questionnaire daily (Everyday group). Both groups completed the questionnaire for a period of 12 weeks. Participants were prompted to complete the FESS questionnaire via automated email reminders administered through Keap, a customer relationship management platform. Participants in the 3-day group received the email 3 times per week—twice on weekdays and once on a weekend—while participants in the everyday group received an email each day of the week. Participants were trained by CEP staff at participating sites on how to access and complete the questionnaire. Upon completing the questionnaire, participants received their score out of 100. These results could then be discussed with CEP staff as part of the participant’s ongoing support. No additional or study-specific training was provided to CEPs involved in the study regarding diet, sleep, or stress management beyond their standard professional training and scope of practice. CEPs delivered usual care consistent with standard CEP services, primarily focused on exercise assessment, prescription, and delivery. No additional engagement, counseling, or behavioral support related to diet, sleep, or stress was provided outside of routine practice, and any discussions were limited to general advice consistent with relevant Australian guidelines and within CEPs’ scope of practice.

### Assessment

Participants in both groups were required to complete an assessment version of the FESS questionnaire, which was administered via SurveyGizmo (Alchemer, Inc) at 3 time points: prior to commencing the intervention (baseline) and at 6 and 12 weeks after the intervention began. The assessment FESS questionnaire was identical to the intervention version, except that it excluded health advice and tips. At each assessment point, participants were asked to complete the assessment questionnaire 3 times within a week.

In addition, participants in the everyday group were invited to wear a GENEActiv triaxial accelerometer (ActivInsights) for 7 consecutive days; participation in this component of the study was voluntary. To minimize the likelihood of user error, participants were provided with at least a 1-week acclimatization period with FESS prior to being issued the accelerometers. A total of 6 GENEActiv accelerometers were used in the study and deployed to participants across 3 rollout rounds. Accelerometers were calibrated to a sampling frequency of 100 Hz for the 7-day data collection period [36]. Devices were programmed to commence recording on the morning of delivery to each participant. Following delivery, participants were encouraged to wear the accelerometer for at least 5 consecutive days, including a weekend day, to optimally capture physical activity. Accelerometer data were then used to calculate total physical activity expressed as counts per minute (CPM).

### Data Analyses

#### Primary Analyses

##### Data Preparation and Processing

FESS assessment questionnaire data were imported and tabulated in Microsoft Excel. Data were first inspected for missing responses. In line with the per-protocol approach, only data for participants who completed one or more FESS assessment questionnaires at each respective time point (ie, 6 and 12 weeks) were included in the analyses for that time point. All categorical responses were converted to numerical values, and the average was calculated for each participant and each question at each assessment point. Details of the conversion process are provided in Multimedia Appendix 1. The converted averaged data were then uploaded to IBM SPSS Statistics (version 30; IBM Corp) for statistical analyses.

##### Observed Changes in Health Behaviors Using the FESS Questionnaire

The 23 FESS items were analyzed individually to explore potential changes in specific health behaviors at 6 and 12 weeks following implementation of the FESS questionnaire. Data were analyzed using Friedman ANOVA. Where appropriate, post hoc comparisons were performed using the Wilcoxon signed-rank test, with Bonferroni correction applied across time-point comparisons for each item.

##### Group Differences in Health Behavior Change

Differences in questionnaire responses from baseline to 6 weeks and from baseline to 12 weeks were calculated for each questionnaire item and each participant. These differences were then analyzed using the Mann-Whitney *U* test.

All inferential statistical analyses were conducted using nonparametric tests due to the primarily categorical nature of the data. Tests were 2-tailed, and statistical significance was set at *P*<.05.

### Secondary Analyses

#### FESS Questionnaire Compliance

FESS questionnaire completion data were extracted from SurveyGizmo and imported into Microsoft Excel. To assess compliance with the prescribed FESS intervention (ie, the completion of intervention FESS questionnaires rather than assessment questionnaires), the proportion of completed questionnaires relative to the total number of prescribed questionnaires was calculated for each participant.

The primary compliance analysis was conducted using an intention-to-treat approach, in which all randomized participants were included in the denominator. Participants who did not complete the prescribed questionnaires were treated as noncompliant. This approach preserves the benefits of randomization, minimizes bias arising from attrition, and provides a conservative estimate of adherence under routine implementation conditions. Compliance proportions were grouped by intervention arm (3-day group and everyday group) and assessment point (6 and 12 weeks), and mean compliance rates were reported.

A second (per-protocol) analysis was conducted to estimate compliance among participants who remained engaged with the study at each assessment time point. In this analysis, participants who did not complete the FESS assessment questionnaire at the 6-week and 12-week assessment time points were excluded from the corresponding analyses (ie, participants who did not complete the FESS assessment questionnaire at 12 weeks were excluded from the 12-week compliance calculation). This analysis provides an estimate of adherence among active participants and allows comparison with the more conservative intention-to-treat estimate to evaluate the impact of attrition on compliance outcomes.

#### FESS Exercise Domain Validity

Accelerometer data were downloaded using GENEActiv PC Software (version 3.2; ActivInsights). Raw data were extracted as .bin files and converted to 60-second epoch .csv files, which were then imported into Cobra (Dr François Fraysse, University of South Australia) running in MATLAB R2019a (MathWorks). A 60-second epoch length was used in accordance with accelerometer processing recommendations for physical activity [36] and to align with the calibration studies underpinning selected physical activity intensity thresholds [37]. Nonwear time was defined as 60 or more consecutive minutes of zero counts [38], and data were manually trimmed to remove these periods. A valid day of accelerometer data required ≥600 minutes of wear time after removal of nonwear periods, with no extreme values (>20,000 CPM) [37]. Established physical activity intensity thresholds were applied to the trimmed periods of active wear time to derive minutes spent in sedentary behavior (SB; <100 CPM) and moderate-to-vigorous physical activity (MVPA; ≥1952 CPM) [37,39]. A valid week required at least 4 days of data, which is consistent with recommendations for reliable weekly physical activity estimations [40]. Data that did not meet these criteria, or accelerometer output rendered unusable due to device error, were excluded from the analyses.

To assess the validity of the exercise domain of the FESS questionnaire, accelerometer data were compared with daily FESS responses for the corresponding period. Specifically, minutes of MVPA recorded by the accelerometer were compared with item 9 of the FESS questionnaire (minutes of at least moderate-intensity exercise [MVE] in the past 24 hours), and accelerometer-measured sedentary time was compared with item 11 (hours spent sitting for more than 15 minutes in the past 24 hours). The proportions of participants who overreported MVE and underreported sitting time relative to accelerometer data were then calculated.

### Ethical Considerations

The study protocol was approved by the Central Adelaide Local Health Network Human Research Ethics Committee (ID: 16356) and the University of South Australia’s Human Research Ethics Committee (ID: 204757), and all participants provided written informed consent prior to participation.

## Results

### Participants

Forty-one participants (23 female) with a median age of 65 (IQR 58‐70) years were assessed for eligibility; all were enrolled in the study, provided consent, and completed the FESS assessment at baseline, with none excluded. Table 1 shows the demographic characteristics of participants by group. Of these, 21 were randomized to the 3-day group and 20 to the everyday group. Not all participants completed the assessment FESS questionnaire at each assessment time point; participant flow is shown in Figure 1.

**Table 1.** Baseline demographic characteristics of participants by group.

Variable	Three-day group (n=21)	Everyday group (n=20)
Age (y), median (IQR)	66 (56‐71)	62 (58‐68)
Male, n	10	8
Female, n	11	12

**Figure 1.**
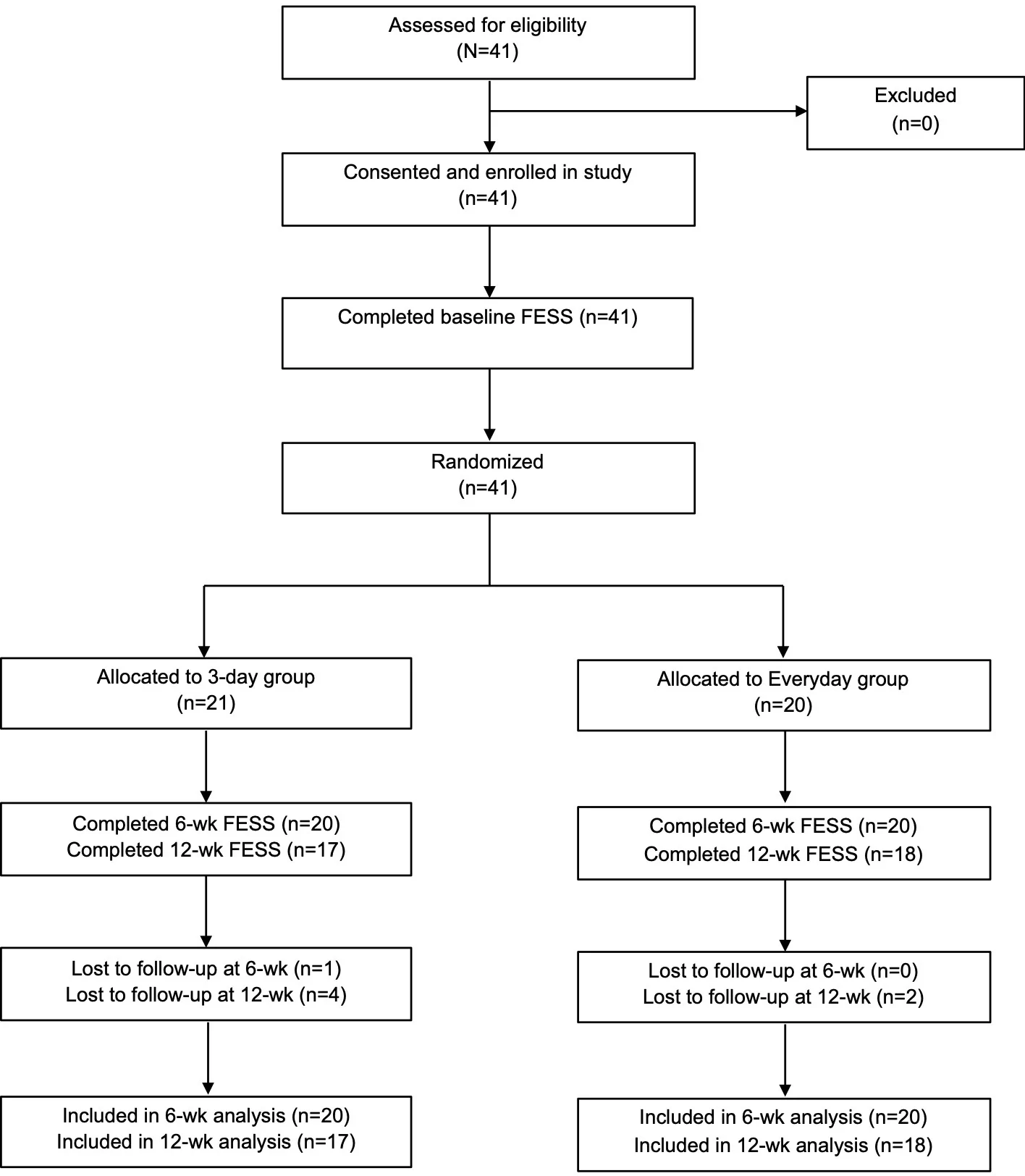
Participant flow through the study. FESS: Food, Exercise, Sleep, and Stress.

### Primary Analyses

#### Observed Changes in Health Behaviors Using the FESS Questionnaire

There was a positive or no change in health behaviors across most items on the FESS questionnaire at both the 6-week and 12-week assessment points, compared with the previous assessment (Table 2). The exceptions were between baseline and 6 weeks and between baseline and 12 weeks for item 12 (strength training); between baseline and 6 weeks for item 15 (mood); and between 6 weeks and 12 weeks for item 16 (recreational activities).

**Table 2.** Descriptive statistics comparing baseline and postintervention outcomes at 6 weeks and 12 weeks.

Item number	Item stem	Measure	Descriptive statistics					
			Baseline (n=41), median (IQR)	6 wk (n=40), median (IQR)	12 wk (n=35), median (IQR)	Directional change in median (baseline-6 wk)	Directional change in median (baseline-12 wk)	Directional change in median (6-12 wk)
Food								
1	How many serves of vegetables in the last 24 h?	Serves	2.5 (2.3‐3.8)	3.6 (2.5‐4.3)	3.8 (3.0‐4.7)	Positive change	Positive change	Positive change
2	How many serves of refined carbohydrates in the last 24 h?	Serves	1.3 (1.0‐2.0)	1.0 (0.9‐1.5)	1.0 (0.4‐1.6)	Positive change	Positive change	No change
3	How many standard drinks of alcohol in the last 24 h?	Standard drinks	0.5 (0.5‐1.5)	0.5 (0.5‐1.8)	0.5 (0.5‐1.2)	No change	No change	No change
4	Consumed fish or nuts in the last 24 h?	Yes/No^a^	1.3 (1.0‐1.7)	1.3 (1.0‐1.5)	1.3 (1.0‐1.8)	No change	No change	No change
5	Consumed processed meats in the last 24 h?	Yes/No^a^	1.7 (1.5‐2.0)	1.7 (1.5‐2.0)	2.0 (1.5‐2.0)	No change	Positive change	Positive change
6	How much water in the last 24 h?	Liters	1.2 (1.0‐1.3)	1.2 (1.1‐1.3)	1.3 (1.2‐1.3)	No change	Positive change	Positive change
7	Consumed sugar sweetened drinks in the last 24 h?	Yes/No^a^	2.0 (1.7‐2.0)	2.0 (1.7‐2.0)	2.0 (1.6‐2.0)	No change	No change	No change
8	Consumed “junk food” in the last 24 h?	Yes/No^a^	2.0 (1.7‐2.0)	2.0 (1.7‐2.0)	2.0 (2.0‐2.0)	No change	No change	No change
Exercise								
9	How many minutes of at least moderate-intensity exercise in the last 24 h?	Minutes	27.5 (20.0‐30.0)	27.5 (21.0‐30.0)	27.5 (19.2‐30.0)	No change	No change	No change
10	Performed high-intensity exercise in the last 72 h?	Yes/No^a^	1.7 (1.3‐2.0)	1.5 (1.3‐2.0)	1.5 (1.2‐2.0)	Positive change	Positive change	No change
11	How many hours sitting for >15 min at a time in the last 24 h?	Hours	6.0 (4.0‐6.7)	6.0 (3.3‐7.3)	6.0 (3.3‐7.0)	No change	No change	No change
12	Performed strength training in the last 72 h?	Yes/No^a^	1.3 (1.3‐1.7)	1.5 (1.3‐2.0)	1.5 (1.0‐2.0)	Negative change	Negative change	No change
Stress								
13	Current level of stress out of 10	1-10^b^	4.5 (2.5‐6.5)	2.8 (1.0‐4.7)	2.5 (1.7‐5.0)	Positive change	Positive change	Positive change
14	How much has stress negatively affected your health?	Less/Same/More^c^	2.0 (1.0‐2.3)	1.5 (1.0‐2.0)	1.3 (1.0‐2.0)	Positive change	Positive change	Positive change
15	In the last 24 h, did you feel reasonably happy?	Yes/Generally/No^d^	1.3 (1.0‐2.0)	1.5 (1.0‐2.0)	1.0 (1.0‐2.0)	Negative change	Positive change	Positive change
16	Taken time out in the last 24 h to do something relaxing or enjoyable?	Yes/No^a^	1.0 (1.0‐1.3)	1.0 (1.0‐1.0)	1.1 (1.0‐1.4)	No change	No change	Negative change
17	Exposed to direct sunlight for ≥20 min or spent ≥60 min outside in the last 24 h?	Yes/No^a^	1.3 (1.0‐1.7)	1.0 (1.0‐1.3)	1.0 (1.0‐1.0)	Positive change	Positive change	No change
18	Smoked a cigarette or exposed to cigarette smoke in the last 24 h?	Yes/No^a^	2.0 (2.0‐2.0)	2.0 (2.0‐2.0)	2.0 (2.0‐2.0)	No change	No change	No change
Sleep								
19	How many hours sleep did you get last night?	Hours	7.3 (6.0‐8.0)	7.7 (7.0‐8.0)	7.7 (7.0‐8.0)	Positive change	Positive change	No change
20	Did you wake from last night feeling rested?	Yes/No^a^	1.3 (1.0‐1.7)	1.0 (1.0‐1.5)	1.0 (1.0‐1.7)	Positive change	Positive change	No change
21	Consumed any form of caffeinated food or beverage after midday?	Yes/No^a^	1.3 (1.0‐1.7)	1.6 (1.0‐2.0)	1.7 (1.0‐2.0)	Positive change	Positive change	Positive change
22	Ate within 30 min of going to bed?	Yes/No^a^	2.0 (2.0‐2.0)	2.0 (1.9‐2.0)	2.0 (2.0‐2.0)	No change	No change	No change
23	Time in front of a screen within 30 min of going to bed?	Yes/No^a^	1.0 (1.0‐1.7)	1.3 (1.0‐2.0)	1.3 (1.0‐2.0)	Positive change	Positive change	No change

a Yes=1; No=2.

b 1=“lowest stress”; 10=“highest stress.”

c Less=1; Same=2; More=3.

d Yes=1; Generally=2; No=3.

The Friedman ANOVA indicated a statistically significant and positive difference in health behaviors over time for item 1 (serves of vegetables), item 6 (liters of water), item 13 (level of stress), item 14 (effect of stress on health), item 17 (exposure to sunlight), and item 19 (hours of sleep; Table 3). Follow-up pairwise comparisons using Wilcoxon signed-rank tests with Bonferroni correction indicated significant changes between baseline and 12 weeks for item 1; between baseline and 12 weeks and between 6 weeks and 12 weeks for item 13; and between baseline and 6 weeks and baseline and 12 weeks for item 19 (Table 3).

**Table 3.** Inferential statistics comparing baseline and postintervention outcomes at 6 weeks and 12 weeks.

Item number	Item stem	Friedman ANOVA (n=35)		Wilcoxon signed-rank test with Bonferroni correction								
				Baseline vs 6 wk			Baseline vs 12 wk			6 wk vs 12 wk		
		Chi-square (*df*)	*P* value	*Z*	*P* value (adjusted)	*r*	*Z*	*P* value (adjusted)	*r*	*Z*	*P* value (adjusted)	*r*
Food												
1	How many serves of vegetables in the last 24 h?	10.5 (2)	.005^a^	−0.443	.19	−0.070	−0.71	.008**^a^**	−0.121	−0.271	.77	−0.046
2	How many serves of refined carbohydrates in the last 24 h?	1.9 (2)	.37	—	—	—	—	—	—	—	—	—
3	How many standard drinks of alcohol in the last 24 h?	0.6 (2)	.73	—	—	—	—	—	—	—	—	—
4	Consumed fish or nuts in the last 24 h?	0.4 (2)	.82	—	—	—	—	—	—	—	—	—
5	Consumed processed meats in the last 24 h?	1.9 (2)	.38	—	—	—	—	—	—	—	—	—
6	How much water in the last 24 h?	7.8 (2)	.02**^a^**	−0.100	>.99	−0.016	−0.500	.11	−0.085	−0.400	.28	−0.068
7	Consumed sugar sweetened drinks in the last 24 h?	0.9 (2)	.61	—	—	—	—	—	—	—	—	—
8	Consumed “junk food” in the last 24 h?	2.2 (2)	.33	—	—	—	—	—	—	—	—	—
Exercise												
9	How many minutes of at least moderate-intensity exercise in the last 24 h?	0.8 (2)	.64	—	—	—	—	—	—	—	—	—
10	Performed high-intensity exercise in the last 72 h?	2.6 (2)	.27	—	—	—	—	—	—	—	—	—
11	How many hours sitting for >15 min at a time in the last 24 h?	0.6 (2)	.71	—	—	—	—	—	—	—	—	—
12	Performed strength training in the last 72 h?	0.7 (2)	.69	—	—	—	—	—	—	—	—	—
Stress												
13	Current level of stress out of 10	26.0 (2)	<.001^a^	0.114	>.99	0.018	1.043	<.001^a^	0.176	0.929	<.001^a^	0.157
14	How much has stress negatively affected your health?	24.6 (2)	<.001^a^	0.029	>.99	0.005	0.786	.003^a^	0.133	0.757	.005^a^	0.128
15	In the last 24 h, did you feel reasonably happy?	2.3 (2)	.31	—	—	—	—	—	—	—	—	—
16	Taken time out in the last 24 h to do something relaxing or enjoyable?	2.1 (2)	.34	—	—	—	—	—	—	—	—	—
17	Exposed to direct sunlight for ≥20 min or spent ≥60 min outside in the last 24 h?	6.5 (2)	.04^a^	0.314	.57	0.050	0.457	.17	0.077	0.143	>.99	0.024
18	Smoked a cigarette or exposed to cigarette smoke in the last 24 h?	2.0 (2)	.37	—	—	—	—	—	—	—	—	—
Sleep												
19	How many hours sleep did you get last night?	9.5 (2)	.009^a^	−0.571	.05^a^	−0.090	−0.586	.04^a^	−0.099	0.014	>.99	0.002
20	Did you wake from last night feeling rested?	0.6 (2)	.71	—	—	—	—	—	—	—	—	—
21	Consumed any form of caffeinated food or beverage after midday?	2.0 (2)	.36	—	—	—	—	—	—	—	—	—
22	Ate within 30 min of going to bed?	0.04 (2)	.98	—	—	—	—	—	—	—	—	—
23	Time in front of a screen within 30 min of going to bed?	5.6 (2)	.06	—	—	—	—	—	—	—	—	—

a Values indicate statistically significant results (*P*<.05).

#### Group Differences in Health Behavior Change

The Mann-Whitney *U* test indicated a statistically significant difference in the change in health outcomes between intervention groups from baseline to 6 weeks for item 13 (level of stress; *U*=273.5; *Z*=1.997; *P*=.046, with a moderate effect size of *r*=0.316); from baseline to 12 weeks for item 14 (impact of stress on health; *U*=214.5; *Z*=2.108; *P*=.04, with a moderate effect size of *r*=0.356); and from baseline to 12 weeks for item 21 (consumed caffeine after midday; *U*=78.0; *Z*=−2.639; *P*=.01, with a moderate-to-large effect size of *r*=0.446). In all cases, the results favored the everyday group (ie, more positive behaviors).

### Secondary Analyses

#### FESS Questionnaire Compliance

FESS questionnaire compliance by intervention arm and assessment point is shown in Table 4. Across both analytical approaches (intention-to-treat and per-protocol), compliance was higher in the 3-day group compared to the everyday group at both assessment points (6 and 12 weeks), and higher at 6 weeks than at 12 weeks within each intervention group. Attrition had only a small impact on compliance outcomes across all analyses, indicating that findings were robust to analytic approaches and to differences in intervention exposure.

**Table 4.** Food, Exercise, Sleep, and Stress (FESS) survey compliance by intervention arm group and assessment point^a^.

Assessment point (wk)	Three-day group		Everyday group	
	Intention-to-treat approach, n/N (%)	Per-protocol approach, n/N (%)	Intention-to-treat approach, n/N (%)	Per-protocol approach, n/N (%)
6	291/378 (77.0)	291/360 (80.8)	584/840 (69.5)	584/840 (69.5)
12	205/378 (54.2)	182/306 (59.5)	428/840 (51.0)	428/756 (56.6)

a The intention-to-treat analysis included all randomized participants (3-day group, n=21); everyday group, n=20) at both assessment points. For the per-protocol analysis, the numbers of participants included were n=20 and n=17 in the 3-day group and n=20 and n=18 in the everyday group at 6 and 12 wk, respectively. Values in the table are presented as n/N (%), where n is the number of compliant questionnaire responses, and N is the total number of possible questionnaire responses.

#### FESS Exercise Domain Validity

Sixteen participants wore an accelerometer, but data from 5 were excluded from the analysis due to device error. Fifty-nine participant-days met the inclusion criteria and were included in the analysis. Of the participant-days analyzed, 3 (5.1%) involved overreporting minutes of at least MVE, and 11 (18.6%) involved underreporting sedentary hours.

## Discussion

### Principal Findings

This study examined changes in health behaviors and compliance across all domains of the FESS questionnaire and assessed the validity of the exercise domain specifically, as an adjunct to services provided by CEPs. The preliminary findings suggest that the FESS questionnaire may be a useful tool for supporting positive changes in health behaviors across several health behavior domains. Differences between groups suggest that the daily use of the questionnaire may encourage stronger behavior change; however, compliance was higher for participants who completed it 3 times per week. Accelerometer data further supported the utility of the tool, with low rates of overreporting exercise and underreporting SB. Taken together, these outcomes highlight the potential of the FESS questionnaire as a low-cost and convenient digital platform that warrants further investigation for the promotion of healthy lifestyle behaviors.

The positive behavior changes observed in the study align with existing evidence that digital interventions can support healthier lifestyle choices [21-24]. In particular, the significant improvements observed in food, stress, and sleep-related behaviors highlight that the digital tool may have supported positive change in these domains, even when delivered alongside indirectly related service (ie, exercise physiology services). Given that most digital health promotion strategies have been found to focus on only one domain of health, typically physical activity, nutrition, or sexual health [41,42], these findings are particularly noteworthy. They demonstrate that a digital behavior change tool addressing multiple domains of health, including those less commonly targeted such as sleep and stress, may be able to be successfully integrated as an adjunct to exercise-focused services, rather than being limited to exercise alone. The reasons underlying these observed outcomes may be due to the design of the intervention itself. The FESS questionnaire’s capacity to prompt reflection and offer practical tips, combined with the opportunity to review results with a CEP, reflects features consistent with established aspects of successful behavior change interventions [32] and may have reinforced positive behaviors and reassured participants that they were meeting healthy behavior guidelines. This reassurance may have alleviated stress, which in turn, could have contributed to improvements in sleep.

Statistically significant changes were observed in a limited number of items (6): serves of vegetables, liters of water, level of stress, effect of stress on health, exposure to sunlight, and hours of sleep. This may reflect the already positive levels of healthy behaviors reported at baseline: most participants consumed a balanced diet, drank alcohol in moderation, did not smoke, and maintained adequate sleep and sleep hygiene practices [43-45]. It is notable, however, that despite the FESS questionnaire being delivered in conjunction with services provided by CEPs, exercise was the one domain where there were no significant improvements. There may be several explanations for this result. First, similar to the other FESS questionnaire domains, the level of exercise undertaken by participants reported at baseline was already high. While direct comparisons between these exercise data and physical activity levels in the general population are complicated by methodological differences, the levels of exercise reported by participants suggest that they would fall within the upper quartile of exercise engagement compared to the general Australian adult population [46]. Second, participants in this study were either a newly referred or existing CEP service client, and therefore, were likely to be on a prescribed exercise program. Taken together, these findings suggest a ceiling effect for healthy behaviors that may have limited the potential for further measurable improvements in these participants.

Another key finding of this study was the contrast between participant compliance and outcomes across the 2 groups. Consistent with previous research [47-49], compliance was lower among those asked to access the FESS questionnaire daily compared to those who accessed it 3 days per week, and it declined in both groups over time. Despite this, there was evidence that more frequent access to the questionnaire was associated with greater positive changes in health behaviors (although it should be noted that only 3 items on the questionnaire reached statistical significance). This aligns with findings from a systematic review and meta-analysis on attrition and adherence in smartphone-delivered interventions for mental health, which examined 10 trials that assessed whether more frequent intervention use was linked to better health outcomes and found mixed results [47]. Taken together, the findings of this study and previous research suggest that while more frequent engagement may have greater potential to promote behavior change, it may be difficult to sustain and result in only moderate additional benefits. Allowing flexibility in frequency of use in this and other digital platforms may therefore be important for optimizing long-term engagement and, hence, behavior change. However, further research is needed to determine the optimal frequency of exposure to digital behavior change platforms.

Finally, we found that the accelerometer data support the validity of the exercise domain of the FESS questionnaire. The relatively low rates of overreporting equal to, or greater than, MVE provide an important first step in validating the tool, given the well-documented inaccuracies of self-report questionnaires, particularly for physical activity [50-52]. In contrast, self-reported SB, consistent with previous research [53], appeared more error-prone, with a higher proportion of participants underreporting sedentary time. It is important to acknowledge, however, that the validation process had limitations: comparisons were limited to 2 items within the exercise domain; the validation involved a small number of participants (n=11); and the accelerometer-derived metrics and the questionnaire responses do not capture the same constructs. These factors may have contributed to discrepancies between the 2 measures.

Additionally, this study has several other limitations. Most notably, the absence of a control group limits the ability to determine whether the observed changes are attributable to the FESS questionnaire itself or simply to routine interactions with a CEP. Convenience sampling may have introduced selection bias [54]. Participants were recruited from CEP clinics and were likely to be more motivated and health conscious than the general population. Eligibility criteria included both newly referred and existing CEP clients, who may differ in motivation, readiness for behavior change, and engagement with services. However, comparative analyses between these 2 subgroups were not performed, and they were treated as a single sample in this study. As such, potential differences in responsiveness to the intervention between newly referred and usual clients cannot be determined. Furthermore, in addition to the potential reporting bias inherent in self-report questionnaires, as highlighted above, participants were encouraged to discuss their behaviors with their CEP, potentially introducing observer bias [55]. Importantly, a similar FESS questionnaire was used both as an intervention tool and as the outcome assessment measure. Repeat exposure to the instrument may have influenced participants’ responses over time, with observed changes potentially partially reflecting increased awareness rather than actual behavior change. However, because the questions have good face validity, being simple reports of easily recalled behaviors, it is unlikely that repeat exposure would change participant reporting. Finally, the intervention of 12 weeks, while adequate for measuring short-term behavior change, is not sufficient to provide insights into the longer-term maintenance of behaviors.

As a proof-of-concept study, these preliminary, hypothesis-generating findings indicate that it is worthwhile to address these limitations by incorporating a control group within a larger, adequately powered randomized controlled trial; recruiting more diverse samples through a multifaceted sampling strategy; conducting subgroup analyses to examine potential differences between newly referred and existing CEP clients; extending follow-up to assess long-term behavior maintenance; and validating the tool across all domains following best practice guidelines [56]. In addition, cost-effectiveness analyses to determine the scalability and economic value of integrating the FESS questionnaire into standard care practices may be warranted.

### Conclusions

This study observed positive changes in multiple health behaviors during the implementation of the FESS web-based questionnaire alongside exercise-focused services. These preliminary, hypothesis-generating findings suggest that CEP services may benefit from embedding structured digital tools to complement traditional face-to-face interactions. By providing education, promoting reflection and accountability between sessions, and addressing a broader range of lifestyle factors, such tools may enhance the care that CEPs deliver. However, larger controlled trials are required to confirm these findings and determine the effectiveness of this approach.

## Supplementary material

10.2196/90115 Multimedia Appendix 1 Food, Exercise, Sleep, and Stress (FESS) questionnaire conversion chart.

10.2196/90115 Checklist 1 CONSORT checklist.

## Abbreviations

CEP: certified exercise practitioner
CONSORT: Consolidated Standards of Reporting Trials
CPM: counts per minute
FESS: Food, Exercise, Sleep, and Stress
MVE: moderate-intensity exercise
MVPA: moderate-to-vigorous physical activity
SB: sedentary behavior
